# Innovative Use of a Coaxial Double-Balloon Catheter System for Treating Gastric Varices

**DOI:** 10.7759/cureus.72543

**Published:** 2024-10-28

**Authors:** Hiroyuki Tokue, Azusa Tokue, Yoshito Tsushima

**Affiliations:** 1 Department of Diagnostic Radiology and Nuclear Medicine, Graduate School of Medicine, Gunma University, Maebashi, JPN

**Keywords:** balloon-occluded retrograde transvenous obliteration, coaxial double-balloon catheter system, gastric varices, gastrorenal shunt, portal hypertension

## Abstract

Herein, we present the case of a 52-year-old female with a history of hepatic surgery who developed gastric varices. Owing to the risk of rupture, a balloon-occluded retrograde transvenous obliteration (BRTO) procedure was performed using a coaxial double-balloon catheter system. Subsequently, the outer balloon catheter was advanced into the gastrorenal shunt. However, venography failed to visualize the varices. Attempts to advance the inner catheter into the varices have been unsuccessful. The inner catheter was advanced into the left gastric vein, establishing a closed circuit around the gastric varices using an outer balloon catheter. A sclerosing agent was subsequently injected through the gap between the outer and inner balloon catheters and via the outer balloon catheter, successfully embolizing the gastric varices. The coaxial double-balloon catheter system is a viable and effective method for treating gastric varices, particularly in cases where traditional BRTO techniques may be challenging owing to their complex vascular anatomy.

## Introduction

Gastric varices are complications of portal hypertension that present a substantial risk of rupture and bleeding and are associated with high mortality rates. Balloon-occluded retrograde transvenous obliteration (BRTO) is a well-established technique for treating gastric varices, particularly in patients with a gastrorenal shunt [1]. However, in complex anatomical situations, modification of BRTO using a coaxial double-balloon catheter system may be required to achieve optimal results. This report presents a unique and successful case of a modified BRTO technique that utilized the gap between the outer and inner balloon catheters of a coaxial double-balloon catheter system.

## Technical report

**Figure 1:**
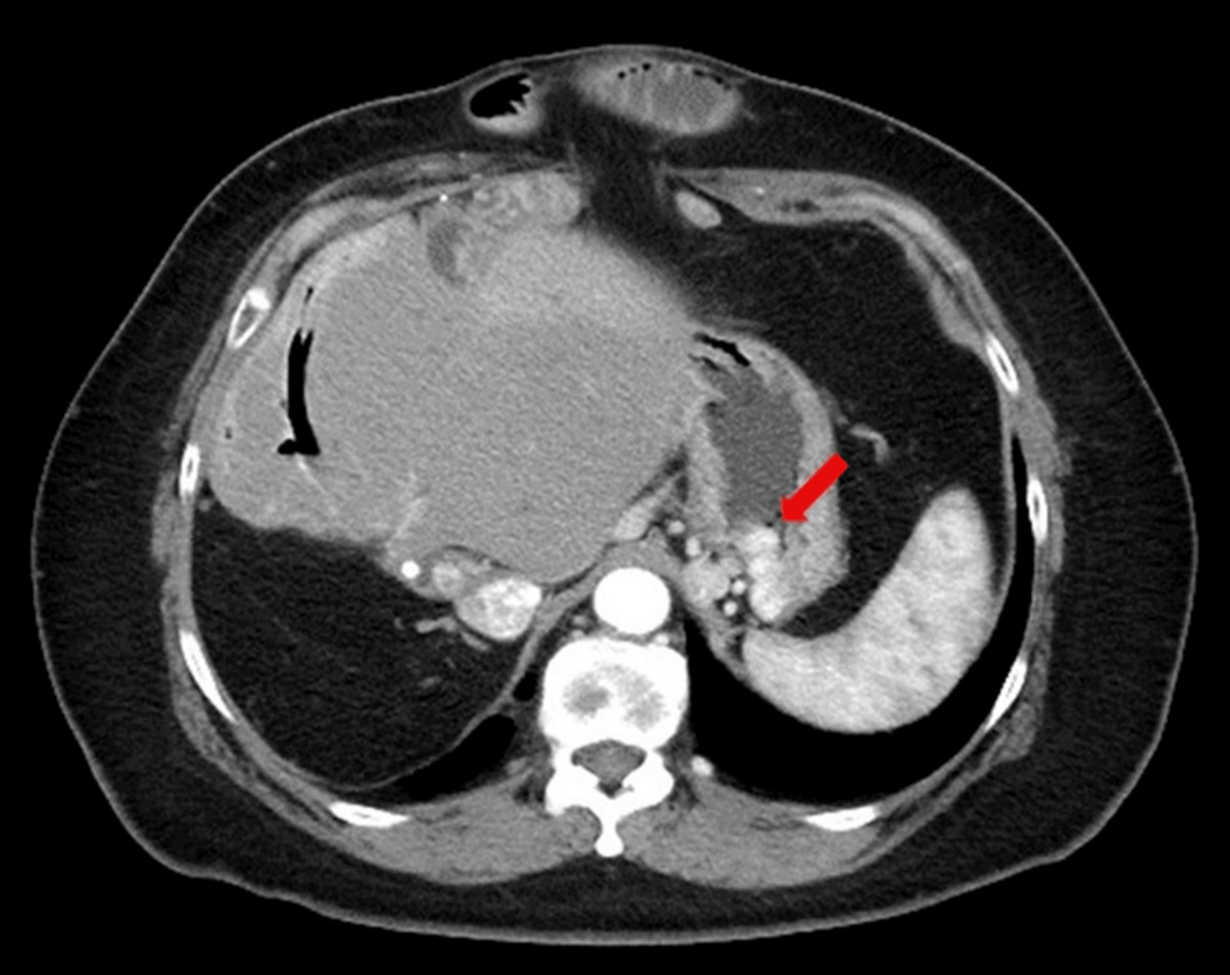
CT imaging demonstrating gastric varices (arrow) in a 52-year-old female with a history of hepatic surgery.

A 52-year-old female with a history of right hepatic lobectomy and choledochoduodenostomy 20 years prior to a traffic accident was referred to our department for the management of gastric varices. Computed tomography (CT) and upper gastrointestinal endoscopy revealed gastric varices with a high risk of rupture (Figure 1). Given the patient’s surgical history and risks associated with varices, BRTO was considered the most appropriate intervention. Other treatment options considered included endoscopic injection sclerotherapy (EIS) and transjugular intrahepatic portosystemic shunt (TIPS). However, EIS was deemed inappropriate due to the presence of a gastro-renal shunt, and TIPS was ruled out due to the patient's history of hepatic surgery.

**Figure 2:**
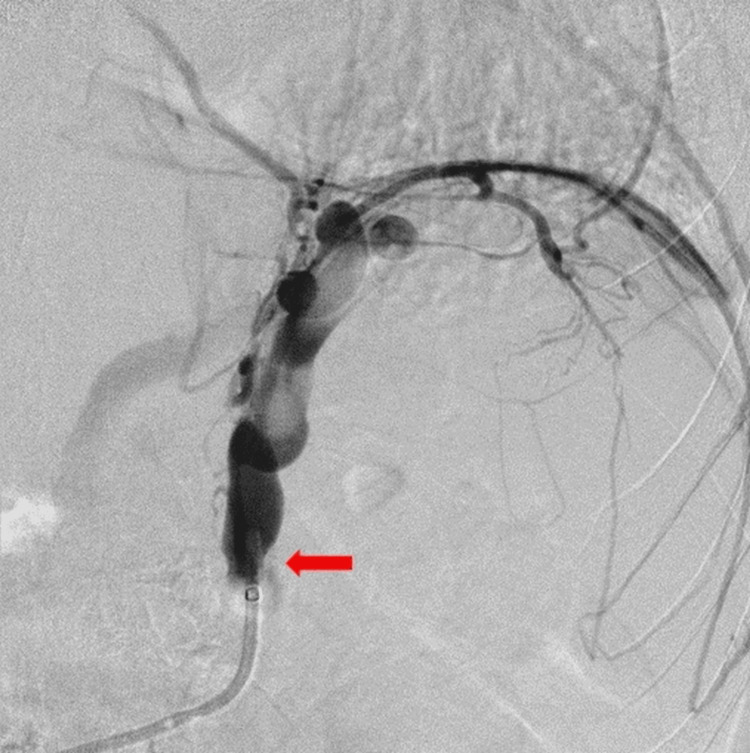
Initial retrograde venography performed with the outer balloon catheter advanced to the left renal vein to access the gastrorenal shunt (arrow; inflate the balloon with carbon dioxide), showing inadequate visualization of the gastric varices.

The procedure was initiated via the right femoral vein using a 10-Fr sheath under local anesthesia. A coaxial double-balloon catheter system (CANDIS: coaxial and double interruption system, Medikit, Tokyo) was utilized, with the outer balloon catheter (9-Fr) advanced into the left renal vein to access the gastrorenal shunt, and retrograde venography was performed. However, visualization of the gastric varices was inadequate (Figure 2).

**Figure 3:**
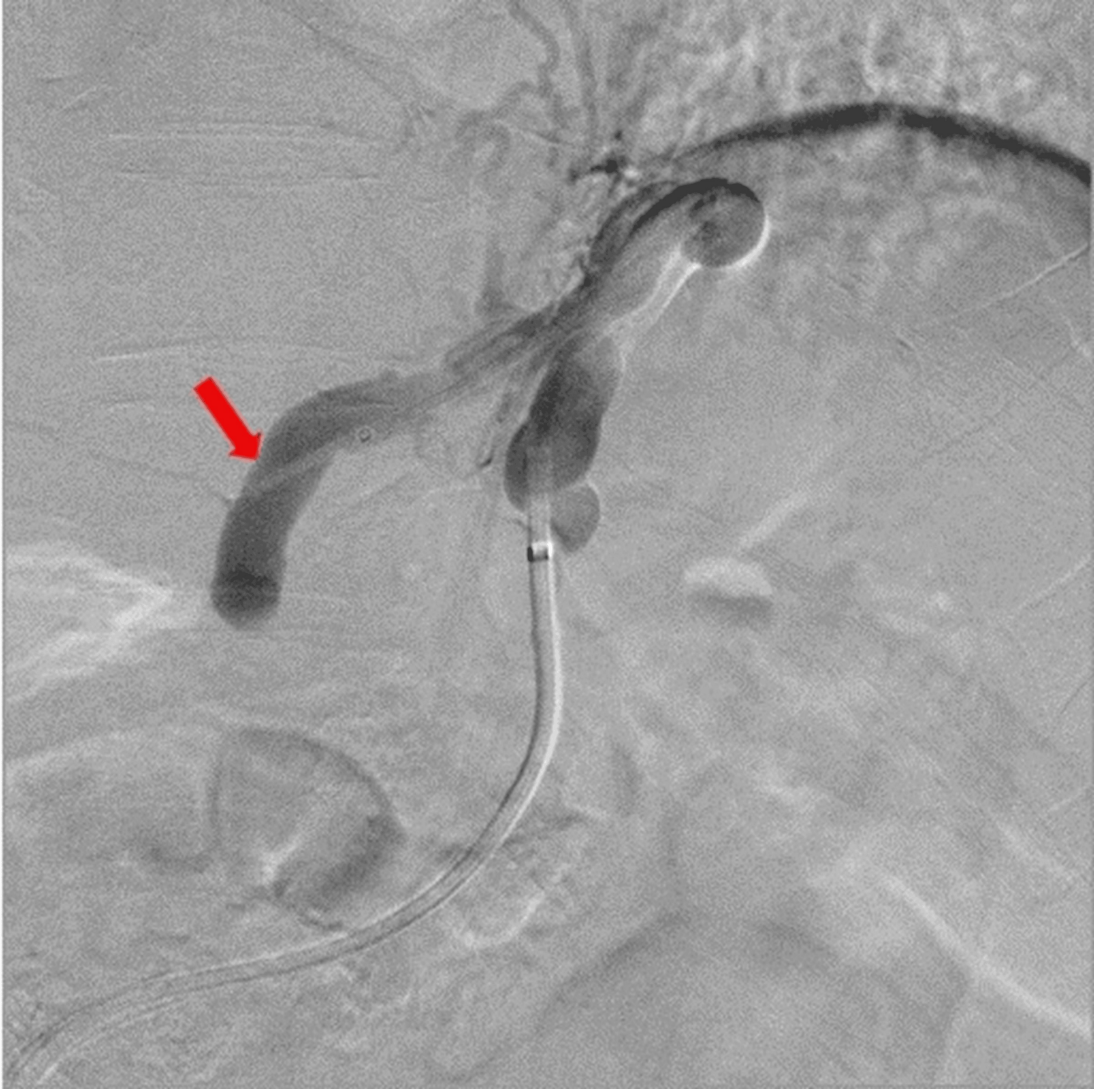
Advancement of the inner balloon catheter toward the left gastric vein (arrow), attempting to enclose the gastric varices.

Subsequently, an attempt was made to advance the inner balloon catheter (5 Fr) to the gastric varices; however, this was unsuccessful, rendering traditional sclerotherapy through the inner catheter unfeasible. The inner balloon catheter (5 Fr) was then advanced toward the supplying vessel (left gastric vein) (Figure 3).

**Figure 4:**
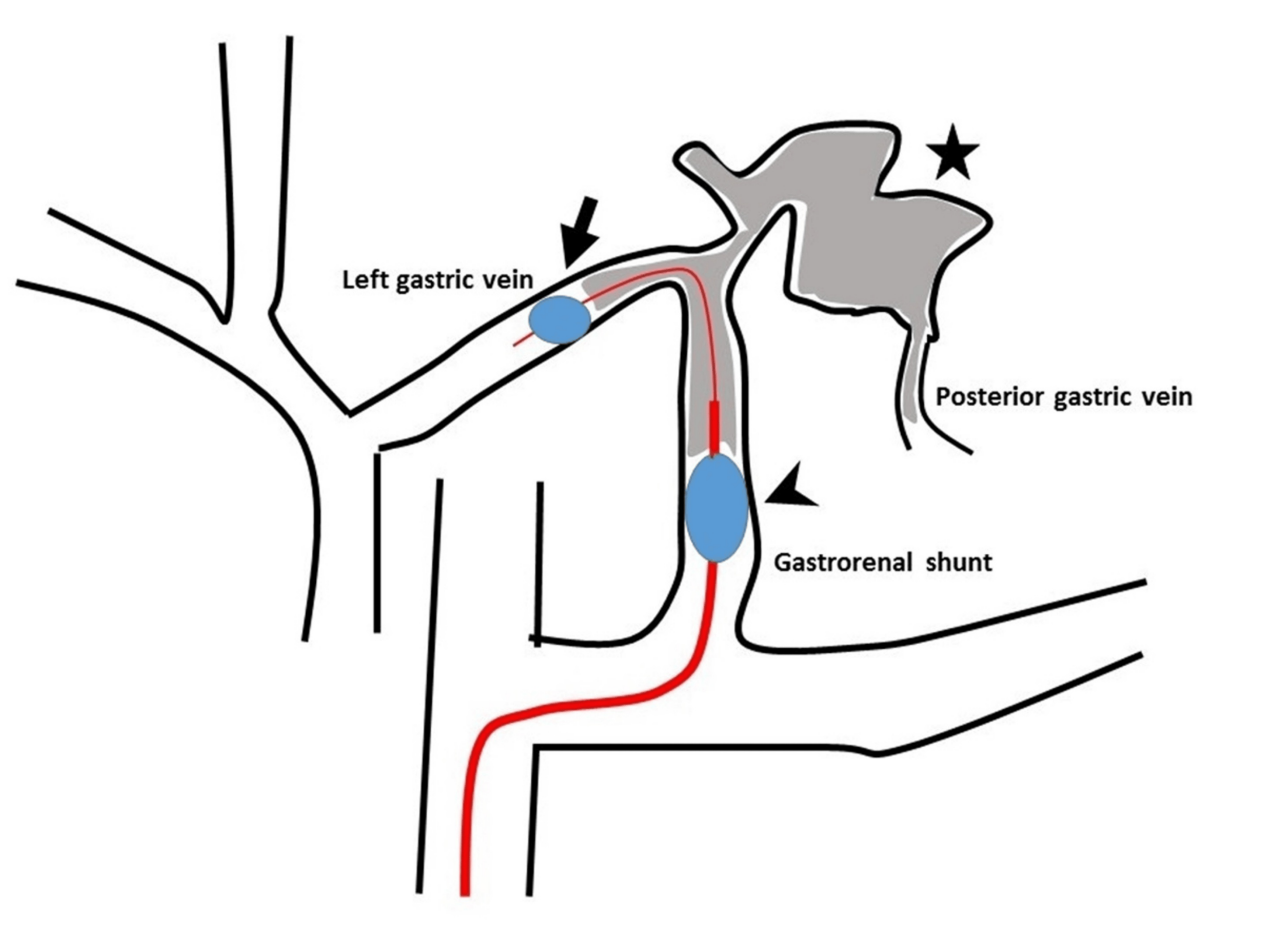
Schematic diagram of a closed circuit created by inner (arrow) and outer balloon catheters (arrowhead) enclosing the gastric varices (star).

The catheters were positioned such that the gastric varices were enclosed between the inner and outer balloons, creating a closed circuit (Figure 4).

**Figure 5:**
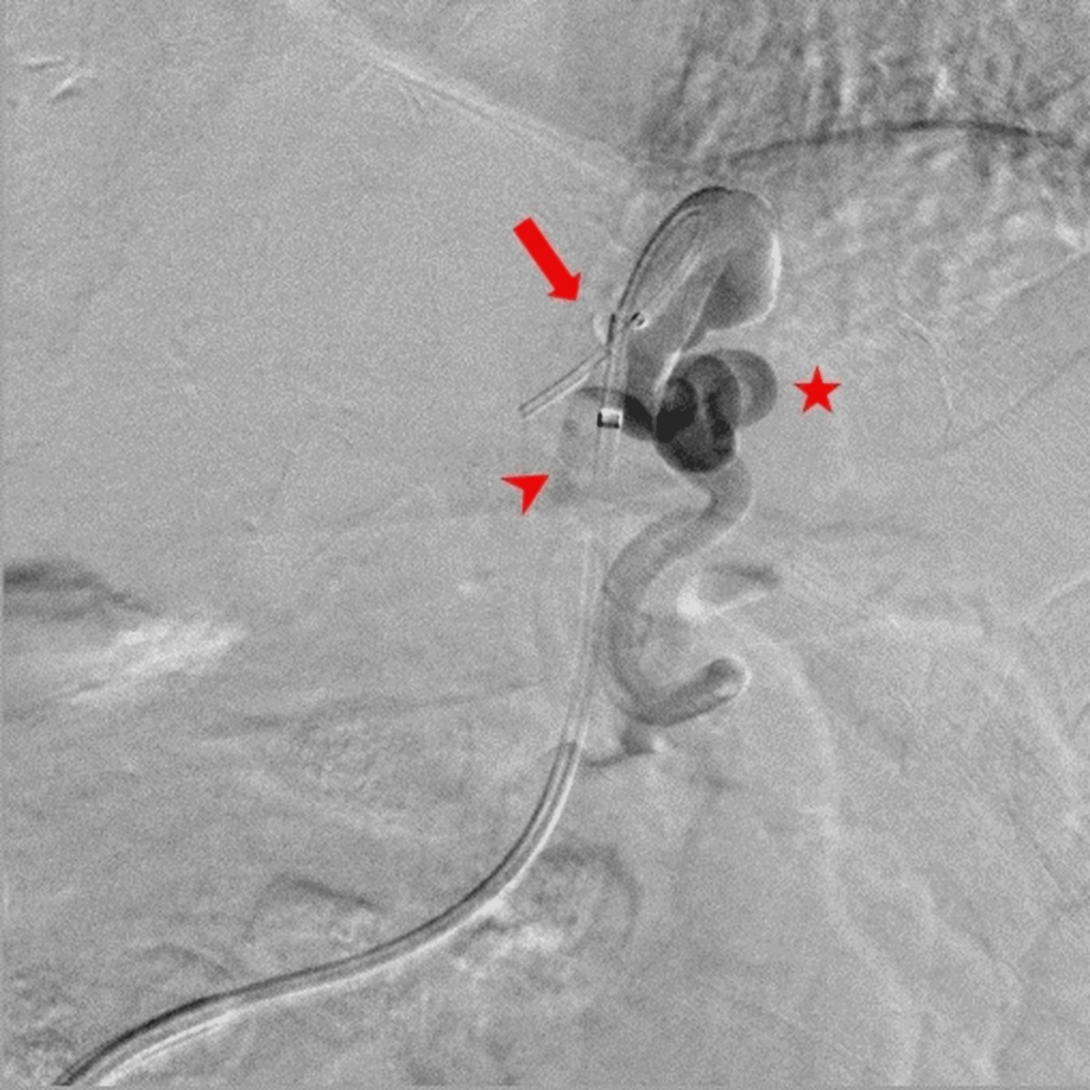
Catheters were positioned so that the gastric varices were enclosed between the inner (arrow) and outer balloons (arrowhead). Contrast medium injected through the outer balloon catheter passing through the gap between the outer and inner balloons, allowing visualization of the gastric varices (star).

There was a gap between the outer and inner balloon catheters. When both balloons were inflated and the contrast medium was injected through the outer balloon, the medium could pass through the gap, allowing visualization of the gastric varices (Figure 5).

**Figure 6:**
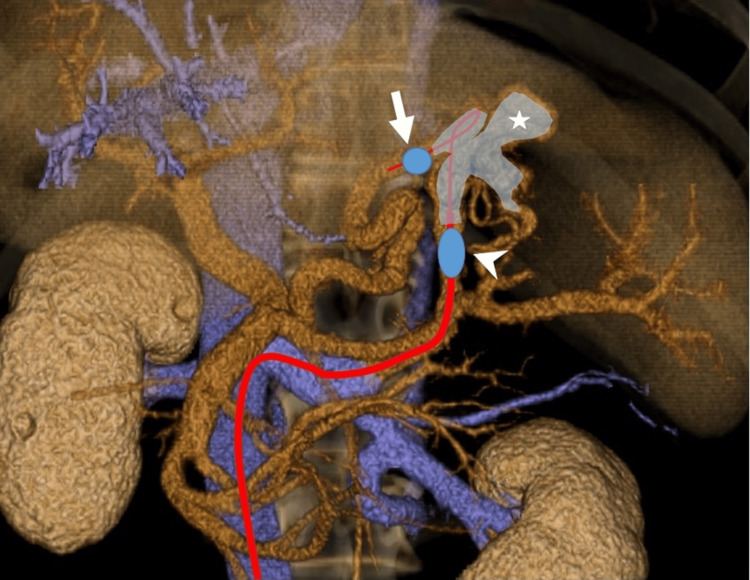
Schematic diagram showing 3D-CT images of a closed circuit created by inner (arrow) and outer balloon catheters (arrowhead) enclosing the gastric varices (star). 3D-CT: three-dimensional computed tomography

Subsequently, 10 cc of the sclerosing agent, ethanolamine oleate iopamidol, diluted to 5%, was injected through the outer balloon catheter. This technique facilitated the effective treatment of gastric varices (Figure 6).

**Figure 7:**
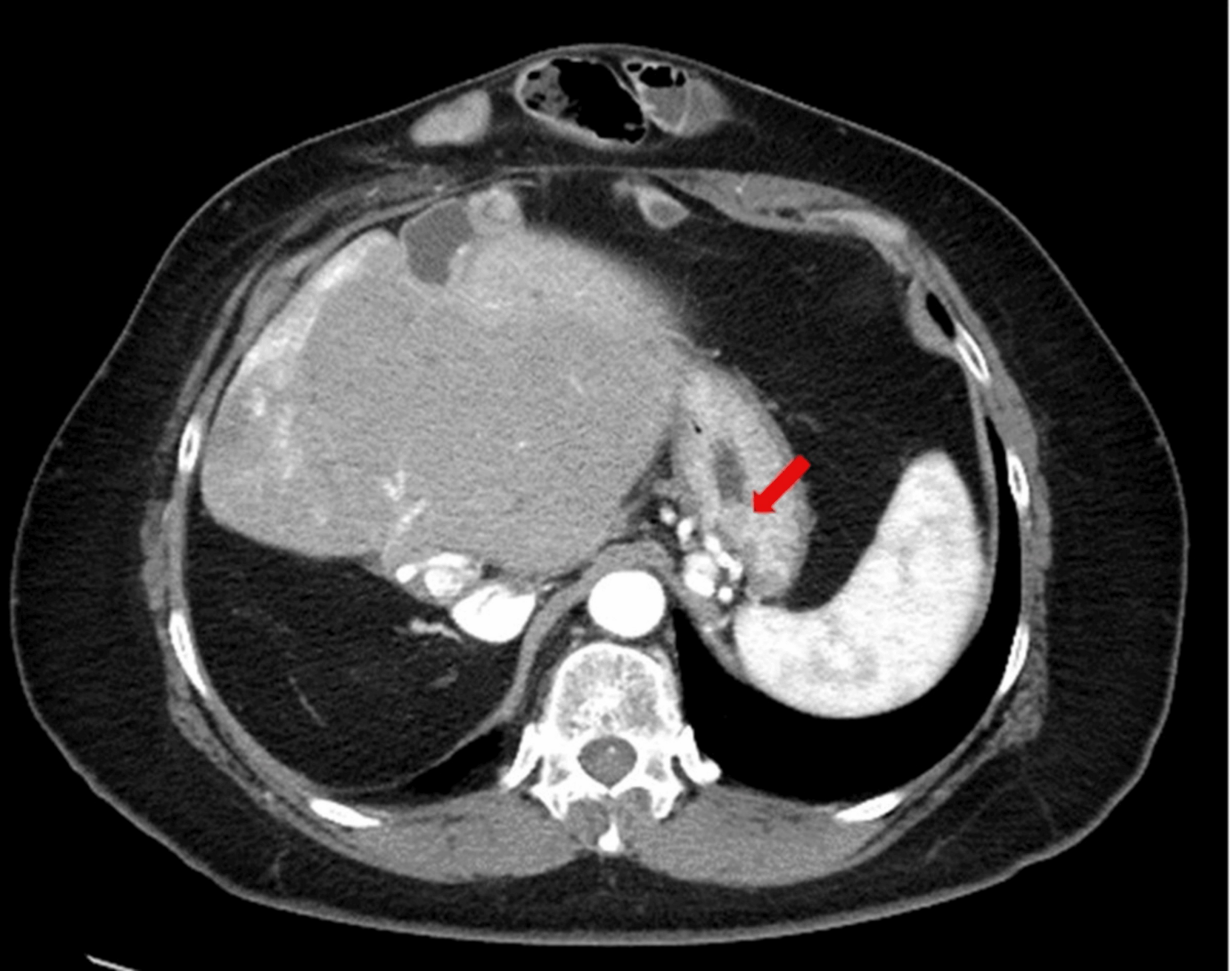
Post-procedure CT image demonstrating complete thrombosis of the gastric varices (arrow) following treatment.

Two days post-procedure, a CT confirmed complete thrombosis of the gastric varices (Figure 7).

## Discussion

Gastric varices, often associated with portal hypertension due to chronic liver disease, exhibit a lower incidence of rupture than esophageal varices (3-30%) but demonstrate a significantly higher mortality rate (45-55%) upon rupture​ [2]. BRTO is a less invasive and highly effective method for treating gastric varices by occluding the outflow route and retrograde injection of a sclerosing agent​ [3].

However, dual balloon-occluded embolotherapy (DBOE), a variation of BRTO, provides an advantage in cases with complex inflow routes, or when varices are poorly visualized on retrograde venography. By occluding both inflow and outflow routes, DBOE simplifies the variceal anatomy, facilitating more effective and complete sclerotherapy. Typically, this method necessitates percutaneous transhepatic puncture of the portal vein, which increases procedural invasiveness compared to BRTO​ [4].

**Figure 8:**
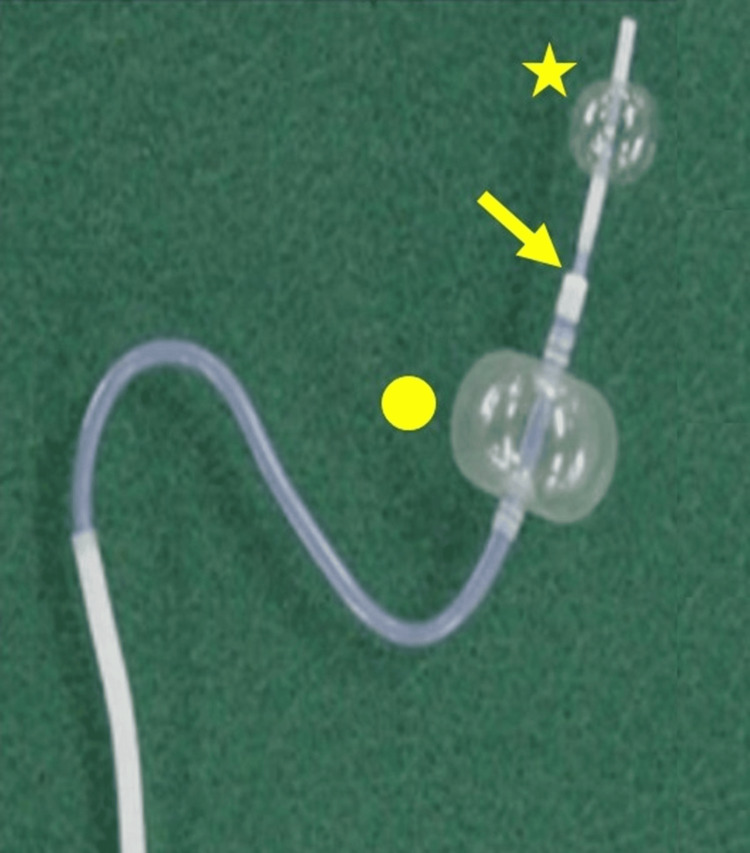
CANDIS used in this study. The inner balloon catheter (star) is coaxially inserted into the outer balloon catheter (circle), creating a gap (arrow) between the two. This gap allowed for the injection of the contrast medium and sclerosing agent. CANDIS: coaxial double-balloon catheter system

In this case, a coaxial double-balloon catheter system (CANDIS) was used to address the challenge of inadequate variceal visualization. The CANDIS was originally developed to allow a catheter to be advanced from a gastrorenal shunt to the vicinity of the varices during the treatment of gastric varices. The CANDIS system comprises an inner balloon catheter (5 Fr), characterized by high flexibility, that can be coaxially inserted into an outer balloon catheter (9 Fr) in advance. The outer balloon catheter, an L- or hook-shaped catheter, is designed to resist kinking owing to its polyamide membrane construction and stainless-wire mesh reinforcement. Balloons made of natural rubber can be inflated to a maximum diameter of 2 cm and 1 cm for outer and inner balloon catheters, respectively [5]. There was a small gap (approximately 0.2 mm) between the outer and inner balloon catheters (Figure 8).

The flexibility and strength of the CANDIS system facilitated successful advancement of the inner balloon catheter to the inflow vessel and creation of a closed circuit, despite the absence of variceal visualization on venography. This setup enabled effective sclerotherapy, resulting in complete thrombosis of the gastric varices.

Limited reports exist on the treatment of gastric varices by creating a closed circuit utilizing a coaxial double-balloon catheter system​ [4]. This case contributes to the growing evidence supporting the utility of the system in complex anatomical situations, particularly where traditional approaches may prove ineffective owing to insufficient visualization of the varices.

The technical success of this procedure highlights the importance of selecting an appropriate catheter system in complex cases. The decision to employ the outer balloon catheter for sclerosing agent injection, a modification necessitated by the gap between the catheters, proved to be effective and may represent a valuable technique in similar cases. Physicians performing BRTO or DBOE should be aware of this option, particularly in cases involving anatomical challenges.

**Figure 9:**
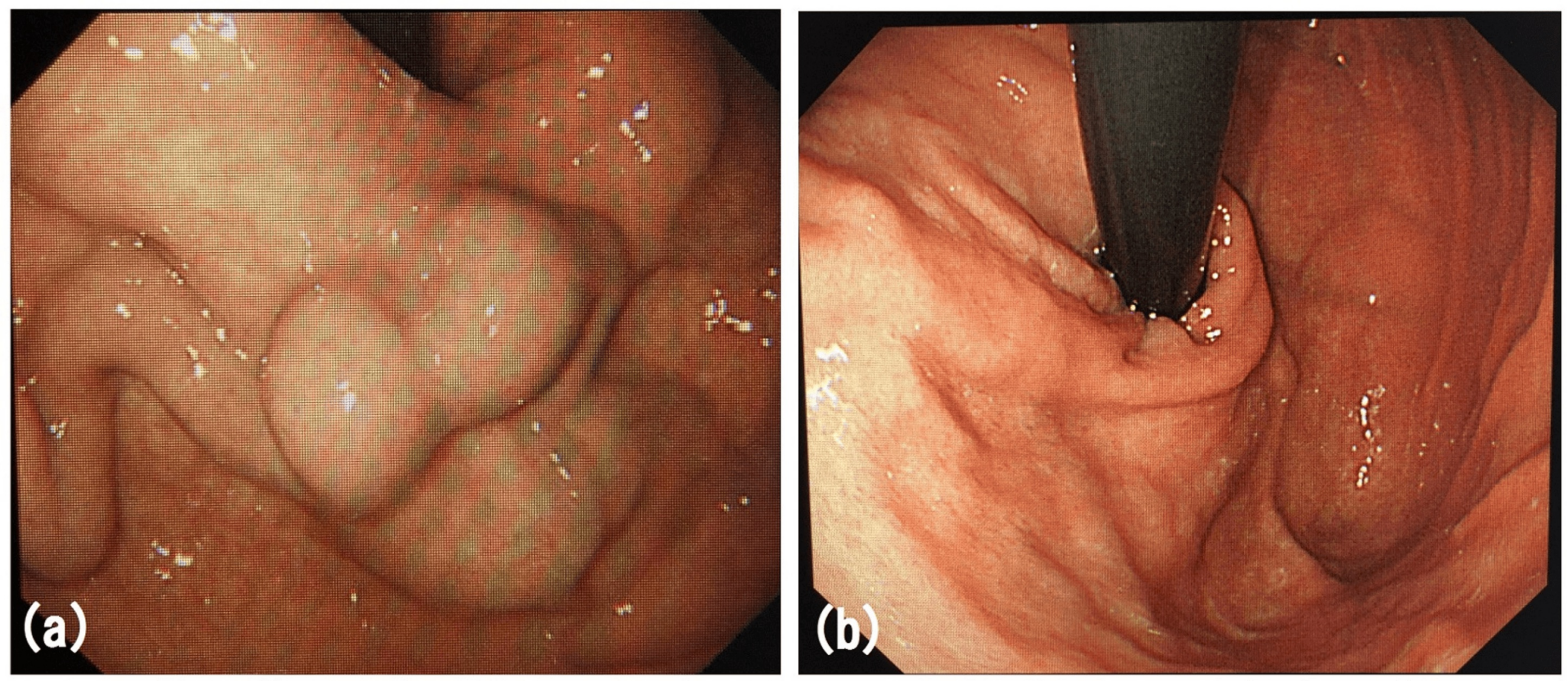
Endoscopy images: (a) Gastric varices were observed before the treatment. (b) No recurrence of gastric varices was observed three years after the treatment.

The patient was followed up for three years after treatment, and no recurrence of gastric varices was observed by contrast-enhanced CT or gastroscopy (Figure 9).

This long-term success highlights the durability of the treatment and effectiveness of the modified BRTO approach using the CANDIS system.

Although this case was successful, it is essential to consider the potential complications associated with the use of the double-balloon system and the modifications described. Possible risks include balloon rupture, migration, and incomplete variceal obliteration if the system is not precisely positioned. In addition, injecting the sclerosing agent through the outer balloon catheter, although effective in this case, may carry the risk of incomplete distribution or unintended embolization if not carefully monitored. Meticulous attention to catheter positioning and careful post-procedure imaging are recommended to minimize these risks.

## Conclusions

The modified DBOE procedure using the CANDIS system is a viable and effective method for the treatment of gastric varices, particularly in cases where traditional BRTO techniques may be challenging owing to their complex vascular anatomy. By utilizing the small gap between the outer and inner balloon catheters to create a closed circuit, this technique enabled precise visualization and successful sclerosis of the varices. The procedure resulted in complete thrombosis of the gastric varices with no recurrence observed over a three-year follow-up. This outcome highlights the importance of selecting an appropriate catheter system tailored to the patient's vascular anatomy to optimize treatment outcomes in complex cases. Further studies are warranted to evaluate the long-term efficacy and safety of this modified approach in a larger cohort.
